# New onset migraine with aura after treatment initiation with ivabradine

**DOI:** 10.1186/1129-2377-14-45

**Published:** 2013-05-29

**Authors:** Till Sprenger, Weera Supronsinchai, Peter J Goadsby

**Affiliations:** 1 Department of Neurology and Division of Neuroradiology, University Hospital Basel, Basel, Switzerland; 2 UCSF Headache Group-Department of Neurology, University of California, San Francisco, San Francisco, CA, USA

**Keywords:** Migraine, Ivabradine, Aura

## Abstract

**Background:**

Migraine with aura is a complex neurological disorder modeled in animals by cortical spreading depression. It is less usual to find complete animal models for the disease so any opportunity to test a human effect back at the bench is welcome.

**Findings:**

We report the case of a 24 year old woman who developed new onset episodic migraine with visual aura shortly after treatment initiation with the I*f* ion channel blocker ivabradine for frequency control in hypertrophic cardiomyopathy. We studied whether ivabradine could alter cortical spreading depression in a suitable animal model. Sixteen rats received either ivabradine or saline, and the number of depolarization shifts and blood flow changes induced by cortical spreading depression were measured in both groups. No significant differences between the ivabradine and saline group were detected.

**Conclusions:**

Ivabradine is an interesting substance since it is known to produce migraine-like phosphenes frequently and the patient we report developed *de novo* migraine with aura. However, we were unable to demonstrate that the drug influences the susceptibility of the brain to cortical spreading depression with acute administration. The combined data show the relationship of migraine aura to cortical spreading depression may have some nuances yet to be identified.

## Findings

### Background

Migraine is a disorder of the brain [1] with recurrent attacks of head pain, photophobia, phonophobia and nausea [2]. About 20-30% of migraineurs also have episodes with aura, typically preceding the head pain and lasting between five and sixty minutes [2]. The most frequent aura symptom is visual disturbance with negative (scotomas) or positive (flickering lights) visual phenomena [3]. Somatosensory, motor or aphasic symptoms are also possible during migraine aura, but less common. The visual changes often propagate throughout the visual field at a speed that has been proposed to reflect cortical spreading depression, a process transiently compromising cortical function [4].

Animal models of migraine are available, but they usually reflect only some aspects of the human condition and additional models are hence useful. We report on the case of a patient with new onset migraine with aura after treatment initiation with the novel ion channel blocker ivabradine, and explore the effect of this drug on the laboratory model of cortical spreading depression.

### Case report

The patient, a 24-year-old Caucasian woman, gave a history of migraine with aura attacks starting about three years prior to review. There was a clear correlation of the onset of migraine with aura with treatment initiation with the I*f* ion channel blocker ivabradine. Before treatment initiation, she had infrequent and low-intensity headaches without typical migrainous features, and without migraine aura, starting in puberty. With ivabradine treatment, she suffered from migraine always with aura at a frequency between once every three months and several times a week. She rated the headache intensity of these attacks as seven to ten out of ten on a verbal rating scale. The pulsating and stabbing pain was usually unilateral in the temporal and parietal as well as periorbital area, more often on the right than on the left. With the episodes she had nausea, photophobia, phonophobia and osmophobia, and no cranial autonomic symptoms. The headache worsened with physical activity. She typically felt very tired during the pain. Migraine triggers included alcohol and potentially high altitude.

All migraine attacks were preceded by visual aura lasting between 10 and 30 minutes. She then saw flickering jagged lines, typically in her left visual hemi-field. These jagged lines developed into growing scotomas during the evolution of the aura. She never experienced somato-sensory, motor or aphasic aura. In addition to the classical aura, the patient also developed phosphenes every night during dim light conditions.

The family history was positive for occasional headaches in her father and infrequent headaches in her mother. Her brother had headaches when drinking alcohol. No clear-cut migrainous features were stated in the family and nobody in the family had aura.

The patient had a history of severe familial hypertrophic obstructive cardiomyopathy (HOCM) and suffered several syncopes and episodes with difficult to control tachycardia. She underwent prophylactic implantation of an automatic implantable cardioverter-defibrillator (AICD) in 2004. The heart condition was well-controlled in terms of frequency control only after ivabradine 5 mg twice daily was added to the preexisting therapy with propranolol 40 mg once a day and verapamil 80 mg twice daily.

We discussed the likely migraine with aura-triggering effect of ivabradine with her cardiologist. However, stopping ivabradine was not an option since it was controlling a potentially fatal heart problem.

The patient had previously also been diagnosed with somnambulism. The patient had a history of infrequent motion sickness and “seeing stars” when standing up.

The neurological exam and a CT scan of the head with contrast including CT venography were unremarkable in the patient. An MRI of the brain could not be performed because of the AICD device.

### Material and methods of the laboratory experiments

#### Surgical preparation and experimental design

All experiments were conducted under license of the University of California, San Francisco Institutional Animal Care and Use Committee and conforming to the National Institutes of Health Guide for the Care and Use of Laboratory Animals.

Male Sprague–Dawley rats (300-380 g) were anesthetized with 60 mg kg^–1^ sodium pentobarbital intraperitoneally (i.p.). The femoral vein and artery were cannulated for fluid administration and monitoring of blood pressure, respectively (CT-1000+ ALM 932, CWE Inc., Ardmore, PA, USA). The rats were maintained anesthetized with a 25–35 mg kg^–1^ h^–1^ infusion of propofol. The tracheal airways were cannulated and ventilated with oxygen enriched air, 2–3 ml, 80–100 strokes min-1 (Small rodent ventilator––Model 683, Harvard Instruments, Kent, UK). End Tidal CO_2_ was monitored (Capstar––100, CWE Inc., Ardmore, PA, USA) and kept between 3.5 and 4.5%. A thermostatically controlled homeothermic blanket system was used to keep the body temperature in physiological range. The blood pressure, end tidal CO_2_, and temperature were electronically displayed. The head of the animal was fixed in a stereotaxic frame. The withdrawal reflex after paw pinch and testing of the corneal reflexes were recorded allowing monitoring of the depth of anesthesia. A midline cutaneous incision was made. A 2 mm craniotomy was performed at the parietal bone (7 mm posterior and 1 mm lateral to bregma) using a saline-cooled dental drill. The dura mater was opened to expose the cortical surface. CSD was induced by cortical application of a KCl pellet (3 mg) on the pia mater.

The rats were separated into two groups. Ivabradine (2 mg/kg, *n* = 8) was given intravenously (i.v.) after the first peak of depolarization shift (DC shift) and cerebral blood flow (CBF) changes induced by CSD. In the control group, rats were injected with saline (0.9%, *n* = 8) intravenously.

#### DC shift and CBF monitoring

Cortical activity was monitored via another craniotomy done 1 mm anterior and 1 mm lateral to the bregma. A glass microelectrode was filled with NaCl 4 M and then an Ag/AgCl wire was inserted perpendicular to the cortex to a depth of 800–1000 μm from cortical surface using a micromanipulator. Another Ag/AgCl electrode was fixed to the nose, which was used as a reference. After the microelectrode was inserted, the fiber optic needle probe of laser Doppler flow meter (Wavelength 780 nm) (VMS-LDF2, Moor Instruments, UK) was placed adjacent to the microelectrode. The tip of the probe was kept at a distance of 1 mm above the cortical surface. All tracings were displayed and saved on a personal computer using an online data analysis system (Spike5, CED UK). The number of peaks of CSD-evoked DC shifts and CBF in the first hour was counted.

### Data analysis

All data are presented as mean ± standard error. Statistical analysis was carried out using SPSS (19.0, IL, USA). The DC shift and CBF were analyzed for possible statistical significance between the ivabradine and control groups using Student’s *t*-test. A value of *P* < 0.05 was considered statistically significant.

### Results of laboratory experiments

End-tidal CO_2_ was 4.64 ± 0.11% (*n* = 8, mean ± SD) and 4.61 ± 0.14% (*n* = 8) in the control and ivabradine groups, respectively.

#### Effect of ivabradine on DC shift and CBF

Topical application of KCl to the cortex triggered a series of DC shifts and cortical hyperemias characteristic of CSD. The number of CSDs was 9 ± 2, and 9 ± 1 in control and ivabradine treated groups, respectively. There was no significant difference between the ivabradine treated group compared with the control group in terms of CSDs (*t*_14_ = 0.0001, *P* = 1.000, Table 1)*.* In addition*,* the amplitude and duration of individual DC shifts was measured. The amplitude of DC shifts was 19.7 ± 3.2 and 21.4 ± 3.8 mV and the duration was 46.8 ± 12.6 and 45.0 ± 15.0 seconds in control and ivabradine treated groups, respectively. The difference was not statistically significant for the amplitude (*t*_14_ = 1.413, *P* = 0.180) and the duration (*t*_14_ = 0.313, *P* = 0.755; Table 1).

**Table 1:** The effect of ivabradine 2 mg/kg i.v. on the depolarization shifts and cerebral blood flow induced by cortical spreading depression (3 mg solid KCl)

	**Control**	**Ivabradine**	**P-value**
	**(*n*** **= 8)**	**(*n*** **= 8)**	
**Depolarization shift**			
- Number of peaks in first hour	9 ± 2	9 ± 1	1.000
- Amplitude (mV)	19.7 ± 3.2	21.4 ± 3.8	0.180
- Duration (s)	46.8 ± 12.6	45.0 ± 15.0	0.755
**Cerebral blood flow**			
- Number of peaks in first hour	11 ± 1	10 ± 1	0.323
- Amplitude (%change from baseline)	198 ± 43	216 ± 40	0.262
- Duration (s)	101.4 ± 47.4	117.0 ± 29.4	0.395

**Figure 1:**
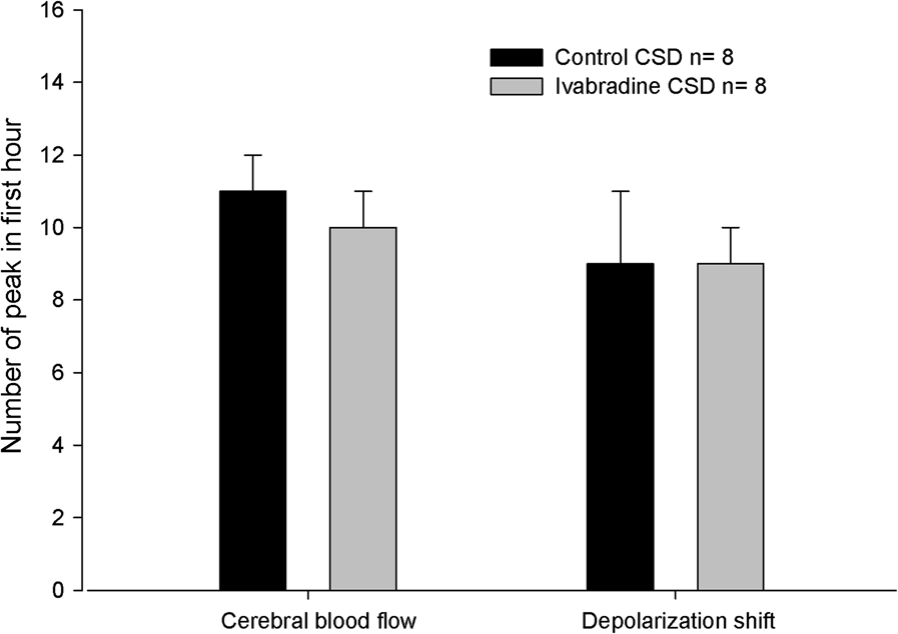
**The effect of ivabradine (2 mg/kg, i.v.) on the number of peak hyperemias in cortical blood flow and the number of depolarization shifts induced by cortical spreading depression.** No significant effects are seen in either measurement.

The number of peak hyperemic events was similar: 11 ± 1, and 10 ± 1 peaks in the first hour in the control and ivabradine treated groups, respectively (*t*_14_ = 1.024, *P* = 0.323; Table 1). The percent change from baseline of peak hyperemia was 198 ± 43 and 216 ± 40 (*t*_14_ = 1.168, *P* = 0.262; Table 1) and the duration of peak hyperemias was 101.4 ± 47.4 and 117.0 ± 29.4 seconds (*t*_14_ = 0.895, *P* = 0.395; Table 1) in the control and ivabradine treated groups, respectively. There was no significant difference between the ivabradine treated group compared with the control group (Figure 1).

#### Effect of ivabradine on cardiovascular response

Ivabradine given intravenously at a dose of 2 mg kg^-1^ significantly decreased the heart rate with a maximum effect of 13% at 60 minutes after application (*F*_2.2,0_ = 15.008, *P* = 0.001; Figure 1). The independent *t*-test showed significant results from 20 minutes (*t*_7_ = 3.297, *P* = 0.013) to 60 minutes (*t*_7_ = 4.637, *P* = 0.002) after injection, when compared with baseline. Ivabradine did not elicit any significant blood pressure changes (*F*_2.13_ = 3.010, *P* = 0.088), nor did the control saline administration (*F*_2.16_ = 1.849, *P* = 0.188).

### Discussion

We report the case of a patient with new onset migraine with typical visual aura after treatment initiation with ivabradine. Ivabradine is a novel I_*f*_ ion channel blocker in sinoatrial node cells and blocker of I_*h*_ channels in the retina and brain. It is indicated for patients with chronic stable angina pectoris due to coronary heart disease, when patients have contraindications for beta-blockers or combination therapy with a beta-blocker is necessary. In our patient, ivabradine was used for frequency control in severe HOCM. There was a clear time-locked relationship between ivabradine treatment start and onset of migraine with aura as well as onset of nightly phosphenes.

A relation between ivabradine and migraine with aura has to our knowledge not been described in the literature, although headache seems to be common (see Ivabradine prescribing information), as with many other drugs. Phosphenes are a well-known side effect of ivabradine occurring in about 15% of patients in the clinical development program [5]. This is thought to be related to its action at the retinal I_*h*_ channels leading to an inhibition of hyperpolarization-activated current. However, a cortical mechanism is possible as the same channels are also abundant in the cortex and thalamus [6,7]. Phosphenes are common, although there is only a low passage of ivabradine across the blood retinal and minimal blood brain barrier passage [8], so a small amount of the drug seems to suffice.

I_*h*_ channels are thought to have a role in modulating neuronal excitability and in light of the clinical observation of new onset migraine with ivabradine, we hypothesized that ivabradine may have an effect on the cortical susceptibility to cortical spreading depression (CSD). As the aura symptoms were lateralized to the left visual hemifield and were quite typical for migraine aura, we assumed the causal mechanism in the patient must be cortical instead of retinal, where one would rather expect monocular visual symptoms or visual symptoms throughout the entire visual field. Interestingly, the animal experiments were unable to demonstrate any effect of ivabridine on CSD. There are several possible explanations for the absence of an effect: 1. the ivabradine effect could be via a different - non-CSD mediated mechanism; 2. the clinical observation could have happened by chance although this seems unlikely given the strong temporal relationship and the known mechanism of action of the drug, as well as the frequent phosphenes in the patient; 3. the intravenous drug dose used in the animal experiment may have been too low to produce a measurable effect as only a small proportion of the drug is crossing the blood–brain barrier [5]. We applied a dose of 2 mg/kg, whereas a typical dose used in clinical practice is 5 mg bid via an oral route. Hence, with an oral bioavailability of about 40% [9], the weight-adjusted dose used in the animal experiment was many times higher than the human dose, making it unlikely that a dosing problem was the reason for the negative results. The dose did alter heart rate by 13%, which suggests it was biologically active. Lastly, it may be that prolonged dosing is required for an effect [10]. This is not the case for topiramate when used at a clinical dose [11] nor with recently identified effects on ASIC channels [12]. This may highlight the extent of what we are yet to learn about CSD. Certainly the ability to use acute administration to screen treatments would be a great advantage.

In conclusion, the clinical case suggests that ivabradine may have facilitatory effects on the development of migraine aura. Such effects may not be directly related to CSD, or may require time to develop. Further knowledge about the exact biological and clinical properties of ivabradine with its more widespread use may help to understand better the frequency of effects on migraine and aura as well as the potential mechanisms. The case serves to remind us that the translation from bench to bedside and back has much to offer and continues to challenge our understanding of migraine mechanisms.

### Consent

Written informed consent was obtained from the patient for publication of this report.

## Competing interest

The authors declared that they have no competing interest.

## Authors’ contributions

TS: contributed the case description and had the original idea of the study, drafted the initial manuscript, reviewed changes by the co-authors and approved the final manuscript. WS: performed the animal experiments and the statistical analysis, commented on the manuscript draft and approved the final manuscript. PJG: was the main supervisor of the project; had the original idea of the animal study, supervised all animal experiments. Read, revised and approved the final manuscript.
